# Neuromuscular Fatigue in Unimanual Handgrip Does Not Completely Affect Simultaneous Bimanual Handgrip

**DOI:** 10.3389/fnhum.2021.763580

**Published:** 2021-11-02

**Authors:** Mikito Hikosaka, Yu Aramaki

**Affiliations:** ^1^Graduate School of Health and Sport Sciences, Chukyo University, Aichi, Japan; ^2^School of Health and Sport Sciences, Chukyo University, Aichi, Japan

**Keywords:** neuromuscular fatigue, unimanual movement, bimanual movement, bilateral deficit, handgrip strength

## Abstract

Simultaneous bimanual movements are not merely the sum of two unimanual movements. Here, we considered the unimanual/bimanual motor system as comprising three components: unimanual-specific, bimanual-specific, and overlapping (mobilized during both unimanual and bimanual movements). If the force-generating system controlling the same limb differs between unimanual and bimanual movements, unimanual exercise would be expected to fatigue the unimanual-specific and overlapping parts in the force-generating system but not the bimanual-specific part. Therefore, we predicted that the decrease in bimanual force generation induced by unimanual neuromuscular fatigue would be smaller than the decrease in unimanual force generation. Sixteen healthy right-handed adults performed unimanual and bimanual maximal handgrip measurements before and after a submaximal fatiguing handgrip task. In the fatigue task, participants were instructed to maintain unimanual handgrip force at 50% of their maximal handgrip force until the time to task failure. Each participant performed this task in a left-hand fatigue (LF) condition and a right-hand fatigue (RF) condition, in a random order. Although the degree of neuromuscular fatigue was comparable in both conditions, as expected, the decrease in bimanual right handgrip force was significantly smaller than those during unimanual right performance in the RF condition, but not in the LF condition. These results indicate that for the right-hand, neuromuscular fatigue in unimanual handgrip does not completely affect simultaneous bimanual handgrip. Regarding the underlying mechanisms, we propose that although neuromuscular fatigue caused by unimanual handgrip reduces the motor output of unimanual-specific and overlapping parts in the force-generating system, when simultaneous bimanual handgrip is performed, the overlapping part (which is partially fatigued) and the bimanual-specific part (which is not yet fatigued) generate motor output, thus decreasing the force reduction.

## Introduction

Simultaneous bimanual movements are not merely the sum of two unimanual movements. When performing symmetrical bimanual movement requiring the simultaneous activation of homologous muscle groups, there are specific interactions between the left and right motor systems (Swinnen, 2002). The interactions have been compared with unimanual movements and/or asymmetrical bimanual movements to investigate various behaviors, including finger movements (Kelso, 1984), hand movements (Spijkers and Heuer, 1995), and reaching (Diedrichsen et al., 2004), as well as their neural basis (Aramaki et al., 2006a,b, 2010, 2011).

Examining this unique difference in neural control between unimanual and bimanual manipulation could be useful for improving sports performance and rehabilitation. Regarding the use of the same limb across unimanual and bimanual movements, Nozaki et al. (2006) reported the existence of unimanual-specific, bimanual-specific, and overlapping motor memories stored in both unimanual and bimanual modes in the acquisition of unimanual and bimanual arm reaching skills. They concluded that unimanual training could break the plateau in performance that typically occurs after extensive bimanual training, which would enable further improvement of bimanual motor performance for arm reaching (Hayashi and Nozaki, 2016). The potential implications of a motor system comprising three components are considerable; however, it remains unclear whether a similar system underlies bimanual voluntary force generation. If three components also exist for maximal voluntary force generation, there may be useful applications in sports training and paretic hand rehabilitation in stroke patients (Kang and Cauraugh, 2018). For example, when an individual’s hand is fatigued by unimanual strength training, switching to bimanual training may enable them to push themselves further in training. Thus, by comparing unimanual and bimanual muscle strength before and after fatigue in unimanual exercise, it may be possible to determine whether the force-generating system comprises three components.

In interactions of simultaneous bimanual control in voluntary force generation, the force generated in the simultaneous bilateral use of two limbs has been reported to reduce performance in each limb in a phenomenon known as the bilateral deficit (BLD) (Henry and Smith, 1961; Ohtsuki, 1981; Škarabot et al., 2016). Henry and Smith (1961) reported a 3% reduction in bimanual handgrip strength compared with unimanual handgrip. Transcallosal interhemispheric inhibition (IHI) is considered to be a major neurophysiological factor in BLD (Oda and Moritani, 1995; Škarabot et al., 2016). Although neuromuscular fatigue that reduces maximal voluntary muscle force (Gandevia, 2001; Taylor et al., 2016) typically occurs in the limb and hemisphere on the exercised side, it may affect the non-exercised limb and the ipsilateral hemisphere. The greater IHI from the hemisphere on the fatigued side to the hemisphere on the non-fatigued side induced by fatigue in unimanual exercise (Bäumer et al., 2002; Takahashi et al., 2009) suggests that unimanual neuromuscular fatigue may have negative effects on simultaneous bimanual movement. Therefore, it is possible that excessive IHI caused by unimanual neuromuscular fatigue leads to impairment of bimanual force generation, thereby increasing the magnitude of BLD.

Meanwhile, if unimanual-specific, bimanual-specific, and overlapping parts exist in the force-generating system, as in motor memory, the properties of neuromuscular fatigue caused by unimanual exercise would be expected to differ from those caused by bimanual exercise. Assuming that the force-generating system controlling the same limb differs between unimanual and bimanual movements, unimanual exercise would be expected to fatigue the unimanual-specific and overlapping parts in the force-generating system but not the bimanual-specific part. Consequently, force reduction in a bimanual task induced by unimanual fatigue may decrease, thereby decreasing the magnitude of BLD as an opposite response to the fatigue-induced imbalance in IHI mentioned above.

Therefore, we sought to investigate whether simultaneous bimanual force generation is advantageous or disadvantageous under conditions of unimanual neuromuscular fatigue. In the present study, we assessed unimanual and simultaneous bimanual handgrip strength before and after a fatiguing task in which submaximal unimanual handgrip strength was maintained until the time to task failure.

## Materials and Methods

### Participants

Sixteen healthy adult men participated in this study (mean ± standard deviation; age: 22.1 ± 1.1 years; height: 171.5 ± 5.0 cm; body mass: 67.2 ± 5.7 kg). We estimated the sample size using G^∗^Power 3.1 with the following parameters: power of 0.95, an α error probability of 0.05, and an effect size of 1.26. The power value of 0.95 was determined by calculating 1.00 – 0.05, to protect from both type I and type II error using the same criteria. The effect size of 1.26 was determined on the basis of our pilot study. The sample size was determined to be 16, considering a potential 20% dropout rate and counterbalancing. Participants were right-handed, and scored between 70 and 100 on the Edinburgh Handedness test (Oldfield, 1971). No participants had a history of neurological or psychiatric disorders, musculoskeletal injury, or neuromuscular disease, and no participants had undergone specific training of the hands or arms. Participants provided written informed consent, in accordance with the Declaration of Helsinki. The experimental protocol was approved by the Human Subjects Committee at Chukyo University Graduate School of Health and Sport Sciences (valid number: 2020-32).

### Experimental Design

To assess changes in force and electromyographic (EMG) activity in unimanual and bimanual handgrip caused by unimanual fatigue, participants performed the maximal handgrip tests before and after the submaximal fatiguing handgrip task. The submaximal fatiguing handgrip task was conducted in the left-hand fatigue (LF) and right-hand fatigue (RF) conditions. The order was randomly selected for each participant.

### Maximal Handgrip Test

The maximal handgrip test was constructed based on our previous study (Hikosaka and Aramaki, 2021). Handgrip force was sampled at 1000 Hz using a grip force transducer (MLT004/ST, ADInstruments, New Zealand) and a data acquisition device (PL3516, ADInstruments). Sampled data were smoothed by an online low-pass filter with a cut-off frequency of 20 Hz using LabChart software (LabChart 8, ADInstruments). Surface EMG signals were recorded from the flexor digitorum superficialis using a wireless EMG sensor (pico, cometa, Italy) during the maximal handgrip test. Disposable Ag/AgCl electrodes were placed slightly ulnarly on the line between the oblique line of radius and the second middle phalanx at 1/4 from the oblique line of the radius based on the previous study (Kong et al., 2010). The signals were sampled at 1000 Hz using a data acquisition device (PL3516, ADInstruments) and filtered with a band-pass filter (10–500 Hz). Recorded data were rectified and smoothed with a fourth-order zero-lag Butterworth low-pass filter with a cut-off frequency of 10 Hz. Participants were instructed to grip as quickly and strongly as possible when prompted by the instruction “Ready? Go,” then sustained maximal force for 3 s until the instruction “Stop.” The test was counterbalanced to minimize the order effects of unimanual and bimanual tasks. Participants performed two unimanual left (UL) and right (UR) handgrips, and two bimanual (BL, bimanual left; BR, bimanual right) handgrips, respectively. Each task interval lasted 1 min. During the test, participants were seated with their shoulders adducted and neutrally rotated, elbows flexed at 90°, forearms in a neutral position, and wrists between 0° and 30° dorsiflexion and between 0° and 15° ulnar deviation. Participants were instructed to move as little as possible. Handgrip force in each task was determined as an average of the highest value for 3-s contraction in two tests. Furthermore, the BLD in the handgrip was calculated with the following equation: *bilateral deficit (%)* = (*bimanual* - *unimanual)/unimanual* × *100.* The bimanual means bimanual left (or right) handgrip force, and unimanual means unimanual left (or right) handgrip force. The average rectified value (ARV) during the maximal handgrip test was integrated from the onset of a 10% increase in the highest EMG value to the end of a decrease of 10% of the highest EMG value and was standardized by exertion time. Finally, ARV in each task was determined as an average of the value of two tests.

### Submaximal Fatiguing Handgrip Task

**FIGURE 1:**
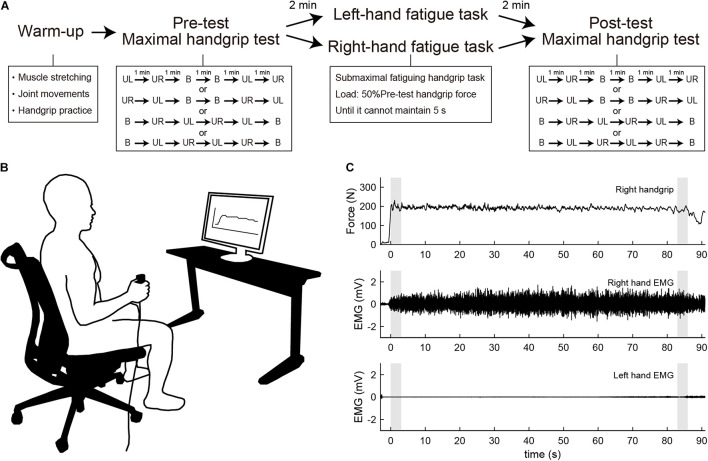
**(A)** Experimental procedure. Participants first performed a warm-up with muscle stretching, joint movements, and handgrip practice. Participants then performed the maximal handgrip test before and after the submaximal fatiguing handgrip task. UL, unimanual left handgrip; UR, unimanual right handgrip; B, bimanual handgrip. **(B)** Experimental setup during the submaximal fatiguing handgrip task. The participant’s handgrip force and the target line for 50% of pre-test unimanual handgrip force were visually fed back *via* a 27-inch display located 1 m in front of the participant. **(C)** Row data traces from a single participant for handgrip force and electromyography (EMG) signal during the right-hand fatigue task. Gray shaded areas indicate the first 3 s and last 3 s of the task. Top, right handgrip force; middle, EMG of right-hand; bottom, EMG of left-hand.

For the submaximal fatiguing handgrip task, data recording was conducted using the same setup as that in the maximal handgrip test. Participants were asked to maintain unimanual handgrip force at 50% of their pre-test unimanual handgrip force. During the fatigue task, each participant’s handgrip force and the target line for 50% of their pre-test handgrip force were visually fed back *via* a 27-inch display located 1 m in front of the participant (Figure 1B). Participants were instructed to assume the same posture of the maximal handgrip test and to keep the opposite hand as still as possible during the task. The fatigue task terminated when participants were unable to maintain a force above 50% of their pre-test handgrip force for more than 5 s, and the time taken to complete the task was defined as time to task failure (TTF). In the submaximal fatiguing handgrip task, the ARV and median frequency (MDF) were calculated in the first 3 s and last 3 s of the fatigue task to assess the degree of neuromuscular fatigue. ARV during the submaximal fatiguing handgrip task was normalized with respect to the pre-test ARV for each subject. MDF was computed using a fast Fourier transform (FFT) method on the raw data within a specified window (first 3 s and last 3 s). The power spectral density was then determined by squaring the FFT. Figure 1C shows raw data traces from a single participant for handgrip force and EMG signals during the right-hand fatigue task.

### Experimental Procedure

Participants visited the laboratory three times and underwent one familiarization session and two experimental sessions. In the familiarization session, participants received a description of the experiment and practiced maximal handgrip and submaximal fatiguing handgrip tasks. In the experimental sessions, participants first warmed up with muscle stretching, joint movements, and handgrip practice. Specifically, participants performed stretching of the wrist flexor/extensor muscles for 10 s, and wrist flexion/extension and internal/external rotation and finger flexion/extension 10 times, then submaximal unimanual left and right and bimanual handgrips at subjective 70% of maximal effort. Participants then performed the maximal handgrip test as a pre-test measure. After a 2-min rest, participants performed the submaximal fatiguing handgrip task in either the LF or RF conditions. After resting for 2 min from the end of the fatigue task, participants performed the maximal handgrip test as a post-test measure. Figure 1A shows the experimental procedure. The experimental sessions employed a crossover design, and randomization was performed using random number generation. Experimental sessions were separated by 1 week. All experimental sessions were performed in the same time slot to minimize daily variability. Participants were instructed to avoid alcohol for 24 h and to avoid caffeine, medicine, and strenuous exercise for 12 h before each session.

### Data Reliability

To test measurement reliability, we performed paired *t*-tests and intraclass correlation coefficient (ICC) analysis for the pre-test handgrip force (Day 1 vs. Day 2). There were no significant differences in pre-test handgrip force between Day 1 and Day 2 for all tasks (UL, UR, BL, and BR) (all ps > 0.1) and the ICC values ranged from 0.789–0.912, indicating that there was little daily variability.

### Statistical Analysis

The TTFs in the submaximal fatiguing handgrip task were compared using paired *t*-test (LF vs. RF). ARV and MDF during the submaximal fatiguing handgrip tasks were compared between the first 3 s and last 3 s using paired *t*-tests. Paired *t*-tests were used to evaluate the contrast in handgrip force from pre- to post-test for each task.

The post-test handgrip force and ARV were normalized with respect to the pre-test value for each subject to assess the difference in fatigue effects between handgrip mode (unimanual vs. bimanual) or fatigue conditions (LF vs. RF). As an *a priori* comparison, the post-test unimanual and bimanual handgrip force and ARV were compared using paired *t*-tests on the basis of the hypothesis that the effects of unimanual fatigue would differ depending on the handgrip mode. In addition, to evaluate the difference in fatigue effects between conditions, paired *t*-tests were performed on the post-test results for the fatigued side handgrip force and ARV.

Each BLD was compared with zero using one-sample *t*-test because negative values indicate lower bimanual handgrip force compared with unimanual handgrip force. As an *a priori* comparison, paired *t*-tests were performed to assess changes in the BLD across time (pre vs. post) on the basis of the hypothesis. The magnitude of BLD would be expected to change if the effects of unimanual fatigue differed between unimanual and bimanual handgrip force. Furthermore, we performed partial correlation analysis to assess the relationship between left- and right-hand force generating capacity in bimanual handgrip during unimanual fatigue. Partial correlation coefficients were used to determine the relationship between post-test BL and BR handgrip force normalized by pre-test by controlling for UL or UR handgrip force in the LF and RF conditions, respectively. *P*-values from *t*-tests were adjusted by Holm–Bonferroni correction, according to the number of *t*-tests. The effect sizes were computed by dividing the mean difference by the standard deviation (SD), whereby ≥ 0.2 is a small effect, ≥ 0.5 is a moderate effect, and ≥ 0.8 is a large effect (Cohen, 1992). All data are reported as means ± SD. Statistical significance was defined as a < 0.05. Statistical analyses were performed using SPSS version 26.

## Results

### Submaximal Fatiguing Handgrip Task

No difference in TTFs was observed between the LF and RF conditions (*t*[15] = −1.729, *p* = 0.104, *d* = 0.44; Table 1). The ARV in the last 3 s of the fatigue task was significantly larger than that in the first 3 s of the fatigue task in both conditions (LF, *t*[15] = −2.478, *p* = 0.026, *d* = 0.87; RF, *t*[15] = −3.403, *p* = 0.008, *d* = 1.09; Table 1). The MDF in the last 3 s of the fatigue task was significantly lower than that in the first 3s of the fatigue task in both conditions (LF, *t*[15] = 11.761, *p* = 0.002, *d* = 2.18; RF, *t*[15] = 7.693, *p* = 0.002, *d* = 1.56; Table 1). Increased ARV and decreased MDF suggested neuromuscular fatigue (Moritani et al., 1986; Cifrek et al., 2009).

**TABLE 1:** Average rectified value (ARV) and median frequency (MDF) of flexor digitorum superficialis in the first 3 s and last 3 s during the submaximal fatiguing handgrip task and time to task failure (TTF).

	**LF condition**			**RF condition**		
	**First**	**Last**	***P*-value**	**First**	**Last**	***P*-value**
ARV (%Pre)	71.8 ± 14.3	98.0 ± 40.4	0.026	66.5 ± 11.5	93.8 ± 33.5	0.008
MDF (Hz)	109.6 ± 18.5	73.6 ± 14.2	0.002	98.4 ± 24.1	65.8 ± 16.9	0.002
TTF (s)		91.1 ± 26.5			81.8 ± 14.5	0.104

*LF, left-hand fatigue; RF, right-hand fatigue. Data are presented as mean ± SD.*

### Comparison Between Pre- and Post-test Handgrip Force

In the LF condition, the UL and BL handgrip force decreased significantly (UL, *t*[15] = 9.169, *p* = 0.008, *d* = 1.41; BL, *t*[15] = 8.206, *p* = 0.008, *d* = 1.40; Table 2). The UR and BR handgrip force did not decrease significantly in the LF condition (UR, *t*[15] = 2.460, *p* = 0.104, *d* = 0.29; BR, *t*[15] = 2.457, *p* = 0.104, *d* = 0.34; Table 2). In the RF condition, the UR and BR handgrip force decreased significantly (UR, *t*[15] = 6.487, *p* = 0.008, *d* = 1.57; BR, *t*[15] = 5.519, *p* = 0.008, *d* = 1.22; Table 2). The UL and BL handgrip force did not decrease significantly in the RF condition (UL, *t*[15] = 1.945, *p* = 0.142, *d* = 0.19; BL, *t*[15] = 1.440, *p* = 0.171, *d* = 0.23; Table 2).

**TABLE 2:** Handgrip force at pre and post submaximal fatiguing handgrip task.

	**LF condition**			**RF condition**		
**Handgrip force**	**Pre**	**Post**	***P*-value**	**Pre**	**Post**	***P*-value**
Unimanual left (N)	358 ± 42	301 ± 38	0.008	353 ± 41	345 ± 43	0.142
Unimanual right (N)	392 ± 47	378 ± 55	0.104	389 ± 51	316 ± 42	0.008
Bimanual left (N)	357 ± 37	303 ± 41	0.008	347 ± 47	337 ± 45	0.171
Bimanual right (N)	385 ± 48	368 ± 52	0.104	384 ± 50	332 ± 33	0.008

*LF, left-hand fatigue; RF, right-hand fatigue. Data are presented as mean ± SD.*

### Difference in Fatigue Effects Between Unimanual and Bimanual Handgrip Force and Average Rectified Value

**FIGURE 2:**
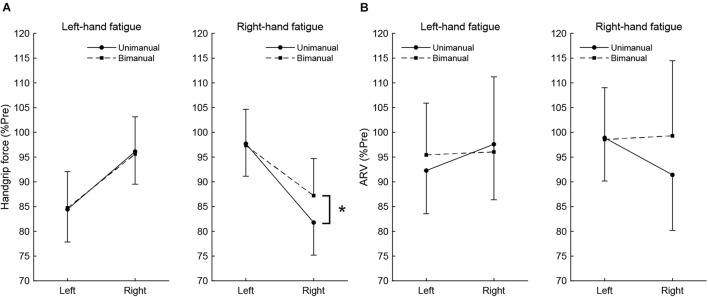
The post-test handgrip force **(A)** and average rectified value (ARV) of flexor digitorum superficialis **(B)**, normalized to pre-test. Circles and linear lines indicate unimanual handgrip task, and squares and dashed lines indicate bimanual handgrip task, respectively. Data are presented as mean ± SD. *p < 0.05.

To assess the difference in fatigue effects between the unimanual and bimanual tasks, the post-test unimanual and bimanual handgrip force values normalized by pre-test values were compared using paired *t*-tests (Figure 2A). In the RF condition, the BR handgrip force was significantly higher than the UR handgrip force (BR, 87.2 ± 8.5 vs. UR, 81.8 ± 10.6%, *t*[15] = −3.198, *p* = 0.036, *d* = 0.57). There was no significant difference between BL and UL handgrip force (BL, 97.7 ± 4.8 vs. UL, 97.3 ± 7.8%, *t*[15] = 0.239, *p* = 1.000, *d* = 0.06). In the LF condition, there were no significant differences between the BL and UL handgrip force (BL, 84.7 ± 7.3 vs. UL, 84.4 ± 6.6%, *t*[15] = −0.174, *p* = 1.000, *d* = 0.04) or the BR and UR handgrip force (BR, 95.6 ± 7.5 vs. UR, 96.1 ± 6.6%, *t*[15] = 0.264, *p* = 1.000, *d* = 0.07).

Similar to handgrip force, the post-test ARV of BR handgrip was higher than the ARV of UR handgrip in the RF condition (BR, 99.3 ± 15.4 vs. UR, 91.4 ± 9.7%, *t*[15] = −2.605, *p* = 0.120, *d* = 0.61), but it was not significant. There was no significant difference between the ARV of BL and UL handgrip (BL, 98.6 ± 8.1 vs. UL, 98.9 ± 6.0%, *t*[15] = 0.179, *p* = 1.000, *d* = 0.05). In the LF condition, there were no significant differences between the ARV of BL and UL handgrip (BL, 95.4 ± 10.5 vs. UL, 92.3 ± 8.7%, *t*[15] = −1.289, *p* = 1.000, *d* = 0.33) or the ARV of BR and UR handgrip (BR, 96.1 ± 15.2 vs. UR, 97.6 ± 11.2%, *t*[15] = 0.486, *p* = 1.000, *d* = 0.12). These results show Figure 2B.

Meanwhile, there were no differences of post-test fatigued side handgrip force and ARV between LF and RF conditions (handgrip force: LF-UL vs. RF-UR, *t*[15] = −1.530, *p* = 0.735, *d* = 0.30; LF-BL vs. RF-BR, *t*[15] = 0.970, *p* = 1.000, *d* = 0.31; ARV: LF-UL vs. RF-UR, *t*[15] = −0.469, *p* = 1.000, *d* = 0.09; LF-BL vs. RF-BR, *t*[15] = 0.925, *p* = 1.000, *d* = 0.29).

### Bilateral Deficit

**FIGURE 3:**
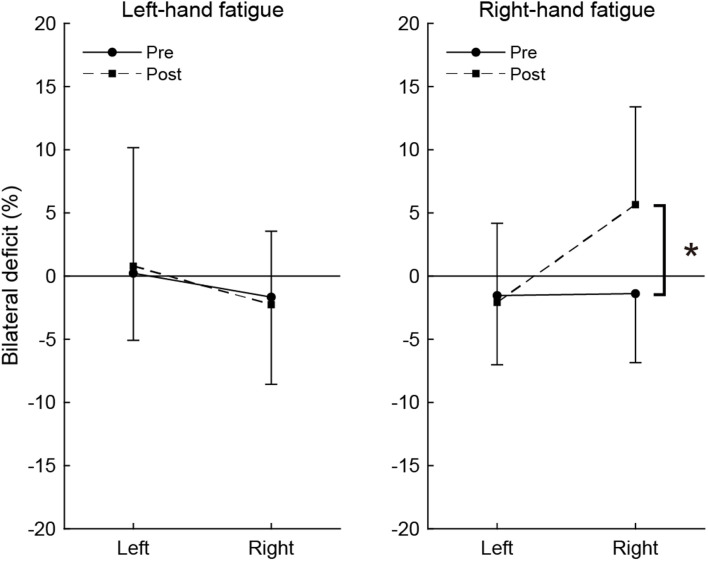
Bilateral deficit in handgrip force. Negative values indicate lower bimanual handgrip force compared with unimanual handgrip force, and positive values indicate higher bimanual handgrip force compared with unimanual handgrip force. Circles and linear lines indicate the values at pre-test, and squares and dashed lines indicate the values at post-test. Data are presented as mean ± SD. *p < 0.05.

Figure 3 shows the magnitude of BLD across pre- and post-test. In the pre-test, the BLD for right-hand was not significant in the RF (−1.39 ± 5.46%, *t*[15] = −1.014, *p* = 1.000, *d* = 0.25) or LF conditions (−1.66 ± 6.90%, *t*[15] = −0.964, *p* = 1.000, *d* = 0.24), but small effect sizes were observed. In the post-test, the bimanual right handgrip force was larger than unimanual right handgrip force in the RF condition and a moderate effect size was observed (5.66 ± 7.75%, *t*[15] = 2.922, *p* = 0.088, *d* = 0.73), even though it was not significant. The BLD for the right-hand in the post-test was not observed in the LF condition (−2.26 ± 5.80%, *t*[15] = −1.555, *p* = 0.987, *d* = 0.39). The BLD for the left-hand in the pre-test was not significant in the LF (0.23 ± 5.31%, *t*[15] = 0.172, *p* = 1.000, *d* = 0.04) and RF conditions (−1.54 ± 5.47%, *t*[15] = −1.125, *p* = 1.000, *d* = 0.28). In the post-test, there were no significant BLD for the left-hand in the LF (0.79 ± 9.38%, *t*[15] = 0.336, *p* = 1.000, *d* = 0.08) and RF conditions (−2.07 ± 6.25%, *t*[15] = −1.327, *p* = 1.000, *d* = 0.33).

To assess the change in BLD induced by the fatigue task, BLD values were compared between pre- and post-test using paired *t*-tests. In the RF condition, there was a significant difference between pre- and post-test in BLD (*t*[15] = −3.038, *p* = 0.032, *d* = 1.49). There was no significant difference in BLD for the left-hand in the RF condition (*t*[15] = 0.323, *p* = 1.000, *d* = 0.13). In the LF condition, there were no significant differences in BLD for the right-hand (*t*[15] = 0.320, *p* = 1.000, *d* = 0.13) or left-hand (*t*[15] = −0.285, *p* = 1.000, *d* = 0.10).

### Partial Correlation of the Left- and Right-Hands in Bimanual Handgrip Force

**FIGURE 4:**
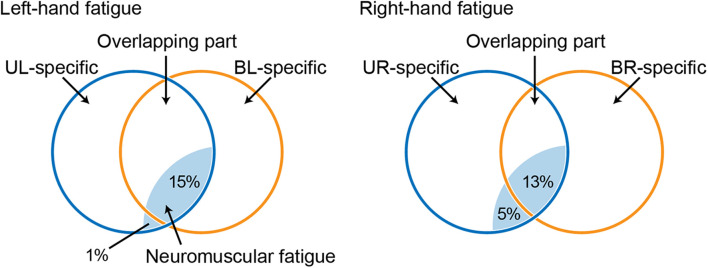
Venn diagram showing the relationship between a force-generating system and unimanual neuromuscular fatigue, based on a previous study (Nozaki et al., 2006; Hayashi and Nozaki, 2016). Circles indicate unimanual-specific (blue) or bimanual-specific (orange) force-generating parts, and the common area of two circles indicates an overlapping force-generating part. Light blue shaded areas indicate neuromuscular fatigue caused by the fatigue task. UL, unimanual left; BL, bimanual left; UR, unimanual right; BR bimanual right.

Partial correlation analysis revealed a significant positive relationship between post-test BR and BL handgrip force normalized by pre-test, controlling for the UR handgrip force in the RF condition (partial *r* = 0.713, *p* = 0.003, Figure 4). Conversely, there was no significant relationship between normalized BL and BR handgrip force, controlling for the UL handgrip force in the LF condition (partial *r* = 0.461, *p* = 0.084, Figure 4).

## Discussion

The present study revealed the effects of neuromuscular fatigue caused by unimanual fatiguing exercise on unimanual and simultaneous bimanual handgrip strength. The results showed that TTF during the submaximal fatiguing handgrip task was similar in the LF and RF conditions. Despite the fact that the degree of neuromuscular fatigue was comparable in the two conditions, the decrease in handgrip strength was smaller for BR than UR handgrip in the RF condition (Figure 2). Furthermore, in the RF condition, the BR handgrip force was greater than the UR handgrip force in the post-test (Figure 3). Reduction of decreased bimanual handgrip strength was observed in the RF condition but not in the LF condition. These results indicate that for the right-hand, neuromuscular fatigue in unimanual handgrip does not completely affect simultaneous bimanual handgrip.

### Reduction in Bimanual Right Handgrip Force and Electromyography Activity Were Less Than Those for Unimanual Right Performance

Unimanual and bimanual handgrip strength on the fatigued side decreased in both the LF and RF conditions (Table 2). Although the degree of neuromuscular fatigue was comparable in both conditions (i.e., no differences in TTF and post-test fatigued side handgrip force between LF and RF conditions), the 13% decrease in BR handgrip force was significantly smaller than the 18% decrease in UR performance in the RF condition (Figure 2). Similarly, the decrease in EMG activity during BR handgrip was smaller than the decrease in EMG activity during UR handgrip and we observed a moderate effect size (*d* = 0.61), but it was not significant. A previous study reported that unimanual neuromuscular fatigue had little effect on bimanual manipulation performance and force coordination (Feeney et al., 2017). In addition, unilateral fatiguing exercise has been reported to reduce unilateral performance significantly more than bilateral performance in the lower limbs (Matkowski et al., 2011). Our results were consistent with these previous studies, which found decreased impairment of bilateral movements under conditions of unilateral neuromuscular fatigue. The current study provided evidence that, although neuromuscular fatigue caused by unimanual handgrip reduces the motor output of unimanual-specific and overlapping parts in the force-generating system, when simultaneous bimanual handgrip is performed, the overlapping part (which is partially fatigued) and the bimanual-specific part (which is not yet fatigued) generate motor output, thus reducing the force reduction (Figure 4).

As in previous reports (Henry and Smith, 1961; Ohtsuki, 1981; Carr et al., 2020), we observed a trend indicating BLD in the only the right-hand results in the pre-test (*d* = 0.24 to 0.25, small effect sizes), whereas the post-test BR handgrip force was greater than UR handgrip force in the RF condition (*d* = 0.71, moderate effect size; Figure 3). Previous studies reported bilateral facilitation, in which bilateral motor performance is greater than unilateral motor performance, in elite weightlifters (Howard and Enoka, 1991) and rowing athletes (Secher, 1975) and a reduction in the magnitude of BLD caused by bilateral training (Häkkinen et al., 1996; Taniguchi, 1997). Furthermore, bilateral strength training has been reported to improve the capability to tolerate fatigue in bilateral performance but not unilateral performance (Rube and Secher, 1991). This phenomenon is considered to reflect the specific adaptation to imposed demands (SAID) principles. We believe that the phenomenon in which BR handgrip force is greater than UR handgrip force can be explained by recruitment of the bimanual-specific part in the force-generating system rather than a response such as bilateral facilitation and SAID principles. This is because this phenomenon is a potential function induced by a transiently fatigued condition, and is not accompanied by changes in neural activity caused by long-term training. These findings support the notion that three components are involved in force generation and suggest that the recruitment of motor systems determines maximal voluntary force.

In most cases, impairments due to peripheral fatigue caused by unimanual exercise, such as the effects of ionic changes on the action potential and the failure of sarcoplasmic reticulum Ca^2+^ release (Allen et al., 2008), are considered comparable in unimanual and bimanual movements. This is because the agonist muscles are considered to be the same for these movements, given that the same effector is used. Meanwhile, central fatigue caused by unimanual exercise, such as inhibition by afferent feedback and decreased motor command from the primary motor cortex (M1) (Gandevia, 2001; Taylor et al., 2016), does not necessarily decrease bimanual movements to the same extent. Assuming that the effects of peripheral fatigue caused by unimanual fatiguing exercise are similar to unimanual and bimanual handgrip tasks, the difference between the decrease in unimanual and bimanual handgrip strength may reflect the effects of central fatigue. Therefore, we speculate that neural activity involved in the bimanual-specific force-generating part may compensate for central fatigue.

### Neural Mechanisms Underlying the Decreased Reduction in Bimanual Right Handgrip Performance

**FIGURE 5:**
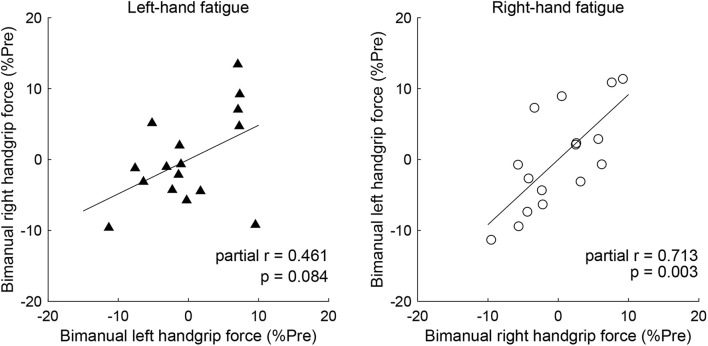
Partial correlation of the left- and right-hand in bimanual handgrip force controlling for unimanual handgrip force. Partial correlation coefficients were used to determine the relationship between post-test bimanual left (BL) and right (BR) handgrip force normalized by the pre-test by controlling for unimanual left (UL) or right (UR) handgrip force in the left-hand and right-hand fatigue conditions, respectively. Data are plotted as residuals of each variable adjusted by control variable. The linear line indicates least squares regression.

**FIGURE 6:**
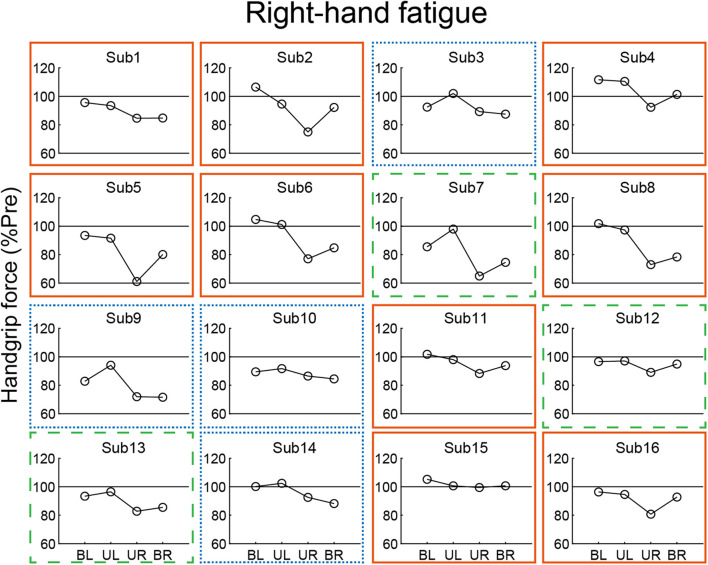
Individual data from post-test handgrip force normalized by a pre-test in the right-hand fatigue condition. Nine subjects showed greater bimanual left (BL) and right (BR) handgrip force compared with unimanual left (UL) and right (UR) performance (orange solid rectangle). Three subjects exhibited greater BR handgrip force than UR performance but lower BL handgrip force than UL performance (green dashed rectangle). Four subjects showed a pattern in which BL and BR handgrip force were reduced more than UL and UR performance (blue dotted rectangle).

In our analysis of the relationship of the left- and right-hand in bimanual handgrip force, a positive correlation was observed between post-test BR and BL handgrip force normalized by pre-test in the RF condition (Figure 5). This finding indicates that left- and right-hand force generation during the bimanual handgrip task were synchronized under conditions of unimanual right-hand fatigue, which suggests that a common motor command was transmitted to both hands. We previously reported that the level of right M1 activity was lower during symmetrical bimanual finger tapping compared with unimanual left finger tapping in right-handed subjects using functional magnetic resonance imaging (fMRI) (Aramaki et al., 2006b). This finding implies that demand for motor commands from right M1 may be reduced by motor commands from left M1 to both hands during bimanual tapping. Furthermore, various studies have reported that the brains of right-handed subjects exhibit structural and functional laterality and that the left hemisphere exhibits greater innervation of the bilateral hands than the right hemisphere, using fMRI (Kim et al., 1993), electroencephalography (Oda and Moritani, 1996), and transcranial magnetic stimulation (TMS) (Ziemann and Hallett, 2001) studies. If the neural control of the bimanual force generation originating from the left M1 is activated by right-hand neuromuscular fatigue, a decrease in bimanual handgrip force may be smaller than a decrease in the unimanual performance for both hands. Analyzing the decrease in handgrip force for individuals revealed that nine subjects exhibited BR and BL handgrip force that exceeded UR and UL performance (Figure 6). For example, motor neurons, which are activated during bimanual movements (Aizawa et al., 1990; Donchin et al., 1998) may play a crucial role in the neural control of bimanual force generation in the left M1. However, seven subjects showed a pattern in which changes in BR and BL handgrip strength were not consistent (Figure 6), which suggests that the reduction of decreased bimanual performance may exhibit individual differences. Thus, the neural control of bimanual force generation does not appear to be determined by a single neural circuit, but the final motor output may be determined as the sum of the neural interaction, such as the ipsilateral motor pathway (Ziemann et al., 1999; Lacroix et al., 2004) from the right hemisphere, greater IHI from the fatigued hemisphere to the non-fatigued hemisphere (Bäumer et al., 2002; Takahashi et al., 2009), or abnormally high IHI from the intact to the lesioned side hemisphere in stroke patients (Murase et al., 2004). To investigate individual differences in the neural control of bimanual force generation, the relationships between behavioral data and neurophysiological parameters should be assessed using fMRI and TMS.

### Laterality of Force-Generating System

In contrast to the RF condition, both UL and BL handgrip force decreased to the same extent in the LF condition (Figure 3). One possibility is that the bimanual-specific part in the force-generating system for the left-hand is small and exhibits extensive overlap (Figure 4). If UL and BL handgrip strength can be reduced by 15% *via* a comparable mechanism, most neuromuscular fatigue would be expected to occur in an overlapping force-generating part. Furthermore, the finding that there was a difference of only 1% between the decrease in UL and BL handgrip strength implies that the fatigue of the unimanual-specific force-generating part was less than that in the RF condition (Figure 4). Thus, right-handed subjects may have had less room to recruit the bimanual-specific force-generating part of the left-hand compared with that of the right-hand. In the context of bimanual manipulation, a previous study reported a right-hand preference for grasping and a left-hand preference for object stabilization among right-handed subjects (Stone et al., 2013). Laterality of hand preference may also influence the recruitment of a bimanual-specific part in the force-generating system. As another possibility, by considering the cross-activation phenomenon that occurs during unilateral contractions (Uehara and Funase, 2014; Cabibel et al., 2020), it may be possible to interpret this asymmetry. Cross-activation (the recruitment of ipsilateral M1 during unilateral contraction) is more pronounced when performed using the non-dominant (left) arm in right-handed subjects (Ziemann and Hallett, 2001), inducing more bilateral brain activation. Assuming a bilateral M1 output motor command for the left hand in both unimanual and bimanual situations may explain the asymmetry in the current results. In any case, the reduction of decreased bimanual handgrip strength may be asymmetric, with a dominant hand-dependent force-generating system in right-handed subjects. However, the present study was limited to data from right-handed subjects only. In addition, EMG activity of BL handgrip appeared to be slightly greater than that of UL handgrip in the LF condition (*d* = 0.33; Figure 2B) and there was a tendency for a relationship between the left- and right-hand in bimanual handgrip force in the LF condition (Figure 5). Thus, further research is needed to clarify the relationship between handedness and the force-generating system.

### Limitations

One limitation of the present study is that although we investigated the effects of unimanual fatigue on bimanual movements, the effects of bimanual fatigue on unimanual movements remain unclear. Further research is needed to comprehensively investigate the effects of bimanual neuromuscular fatigue on unimanual movements and vice versa, to identify unimanual-specific, bimanual-specific, and overlapping parts in the force-generating system. Moreover, unilateral knee extensor fatigue was found to extend the magnitude of the BLD in a previous study (Owings and Grabiner, 1998), which suggests that different responses in the upper and lower limbs are possible. Because the reduction of decreased bimanual motor performance under conditions of neuromuscular fatigue may be a phenomenon specific to the handgrip task, this finding should be interpreted carefully. In addition, because it was not possible to measure the degree of neuromuscular fatigue in the present study, we were unable to detect where neuromuscular fatigue originated. Therefore, our discussion regarding the neural mechanisms of force-generation remains speculative. Neurophysiological studies, such as voluntary activation using TMS of M1 and twitch interpolation using peripheral electrical stimulation, will be needed to verify the details of neuromuscular fatigue.

## Conclusion

Although right-hand neuromuscular fatigue decreased right handgrip strength and EMG activity, the decrease in bimanual right handgrip strength was smaller than those for unimanual right handgrip performance. In addition, bimanual right handgrip strength was greater than unimanual right handgrip strength under conditions of unimanual right-hand fatigue. These results indicate that for the right-hand, neuromuscular fatigue in unimanual handgrip does not completely affect simultaneous bimanual handgrip. Regarding the underlying mechanisms, we propose that although the unimanual-specific and overlapping parts in the force-generating system are fatigued by unimanual fatiguing exercise, a simultaneous bimanual task may attenuate force reduction because the non-fatigued bimanual-specific part generates the motor output.

## Data Availability Statement

The data that support the findings of this study are available from the corresponding author, YA, with permission from our ethical committee.

## Ethics Statement

The studies involving human participants were reviewed and approved by the Human Subjects Committee at Chukyo University Graduate School of Health and Sport Sciences. The patients/participants provided their written informed consent to participate in this study.

## Author Contributions

MH and YA designed the study, analyzed the measured data, and wrote the manuscript. MH performed the measurements. Both authors contributed to the article and approved the submitted version.

## Conflict of Interest

The authors declare that the research was conducted in the absence of any commercial or financial relationships that could be construed as a potential conflict of interest.

## Publisher’s Note

All claims expressed in this article are solely those of the authors and do not necessarily represent those of their affiliated organizations, or those of the publisher, the editors and the reviewers. Any product that may be evaluated in this article, or claim that may be made by its manufacturer, is not guaranteed or endorsed by the publisher.
