# Corrigendum: Neuromuscular Fatigue in Unimanual Handgrip Does Not Completely Affect Simultaneous Bimanual Handgrip

**DOI:** 10.3389/fnhum.2022.962181

**Published:** 2022-07-06

**Authors:** Mikito Hikosaka, Yu Aramaki

**Affiliations:** ^1^Graduate School of Health and Sport Sciences, Chukyo University, Aichi, Japan; ^2^School of Health and Sport Sciences, Chukyo University, Aichi, Japan

**Keywords:** neuromuscular fatigue, unimanual movement, bimanual movement, bilateral deficit, handgrip strength

In the original article, there were errors. In this paper, we have referred to the work by Kelso (1984) as an example of “tapping” and the work by Spijkers and Heuer (1995) as an example of “drawing” in the introduction section. However, their works did not involve “tapping” and “drawing” as we report, but rather free movements of the index fingers and hand movements, respectively. Thus, we need to change the term “tapping” to “finger movements” and the term “drawing” to “hand movements”.

A correction has been made to the Introduction, Paragraph 1:

Simultaneous bimanual movements are not merely the sum of two unimanual movements. When performing symmetrical bimanual movement requiring the simultaneous activation of homologous muscle groups, there are specific interactions between the left and right motor systems (Swinnen, 2002). The interactions have been compared with unimanual movements and/or asymmetrical bimanual movements to investigate various behaviors, including finger movements (Kelso, 1984), hand movements (Spijkers and Heuer, 1995), and reaching (Diedrichsen et al., 2004), as well as their neural basis (Aramaki et al., 2006a,b, 2010, 2011).

The authors apologize for this error and state that this does not change the scientific conclusions of the article in any way. The original article has been updated.

## Publisher's Note

All claims expressed in this article are solely those of the authors and do not necessarily represent those of their affiliated organizations, or those of the publisher, the editors and the reviewers. Any product that may be evaluated in this article, or claim that may be made by its manufacturer, is not guaranteed or endorsed by the publisher.
